# Next-generation sequencing reveals clinical features and prognosis of gene mutations in Chinese children with T-cell acute lymphoblastic leukaemia

**DOI:** 10.3389/fonc.2025.1666527

**Published:** 2025-09-26

**Authors:** Senlin Zhang, Xinran Chu, Zhiheng Li, Qi Ji, Na Meng, Wei Gao, Xiao Zhang, Yixin Hu, Li Gao, Bohan Li, Ping Chen, Huihui Wan, Yu Liu, Yongping Zhang, Yuanyuan Tian, Shuiyan Wu, Yizhen Li, Shaoyan Hu, Hu Liu

**Affiliations:** ^1^ Department of Hematology and Oncology, Children’s Hospital of Soochow University, Soochow University, Suzhou, China; ^2^ Department of Pediatrics, Xinxiang Central Hospital, The Fourth Clinical College of Xinxiang Medical University, Xinxiang, China; ^3^ Department of Hematology, Children’s Hospital of Shandong University, Jinan, China; ^4^ Suzhou Jsuniwell Medical Laboratory, Suzhou, China; ^5^ Pediatric Translational Medicine Institute, Shanghai Children’s Medical Center, School of Medicine, Shanghai Jiao Tong University, National Health Committee Key Laboratory of Pediatric Hematology and Oncology, Shanghai, China; ^6^ Jiangsu Pediatric Hematology and Oncology Center, Suzhou, China; ^7^ Hematologic Intensive Care Unit, Children’s Hospital of Soochow University, Suzhou, China; ^8^ Pediatric Hematology and Oncology Key Laboratory of Higher Education Institutions in Jiangsu Province, Suzhou, China

**Keywords:** T-cell acute lymphoblastic leukaemia, gene mutations, paediatric, next-generation sequencing, prognosis

## Abstract

**Background and objective:**

The 5-year overall survival (OS) for paediatric T-cell acute lymphoblastic leukaemia (T-ALL) exceeds 80% under the current treatment strategies; however, some patients suffer from treatment failure. Next-generation sequencing (NGS) identified recurrent mutated genes in T-ALL that might affect diagnosis, classification, prognostic stratification, and treatment response. This study aimed to characterise the clinical features and prognosis of gene mutations in paediatric patients with T-ALL.

**Methods:**

We enrolled 144 paediatric patients with T-ALL at our centre. Chi-square or Fisher’s exact tests were used for categorical variables, and Kaplan–Meier and log-rank tests analysed the survival rates of these patients.

**Results:**

The most common mutations were in *NOTCH1* (58.3%), *FBXW7* (19.4%), and *PTEN* (17.4%). Of 1262 gene mutations detected, 50 had a mutation frequency of >1%. Common mutations were not correlated with 5-year OS. Patients with higher *NOTCH1* mutation loads had a lower proportion of D15 minimal residual disease ≥0.01% and better survival than those with a lower load.

**Conclusion:**

This study reported the gene mutation spectrum of Chinese paediatric T-ALL, highlighting the role of NGS in molecular classification, risk stratification, and prognosis. Additionally, we emphasised the role of the variant allele frequency of *NOTCH1* mutations in the treatment response and prognosis of childhood T-ALL.

## Introduction

T-cell acute lymphoblastic leukaemia (T-ALL) is an aggressive hematologic malignancy characterised by the abnormal presence of immature T-cell progenitors, representing approximately 15% of paediatric ALL cases (1, 2). This incidence is generally consistent across different regions, including China (3). The 5-years overall survival of paediatric patients with T-ALL exceeds 80% under current treatment strategies; however, some patients experience relapse and drug resistance, which leads to treatment failure (4, 5). Previously, gene mutations have been associated with risk stratification, treatment response, and prognosis in paediatric patients with T-ALL (6, 7). The *NOTCH1* or *FBXW7* mutation is the most common in T-ALL and has been linked to a better outcome (8–10). However, Zuurbier et al. indicated that the *NOTCH1* or *FBXW7* mutation could predict a better prednisone response but did not influence the prognosis of pediatric T-ALL (11). Alterations in other genes such as PHF6 and LEF1 have also been reported in paediatric T-ALL, although their prognostic significance remains controversial. While some smaller studies have suggested that PHF6 or LEF1 abnormalities may be associated with inferior outcomes, more recent evidence from larger cohorts indicates that PHF6 mutations may be linked to favourable prognosis (12, 13). Therefore, the clinical relevance of these less common genetic alterations requires further clarification. Several studies have explored the relationship between gene mutations and the clinical characteristics and prognosis of paediatric T-ALL; however, the conclusions have been inconsistent and require further investigation. Here, we report the results of gene mutation detection through next-generation sequencing (NGS) and the characteristics and conditions of survival in 144 paediatric patients with T-ALL. Additionally, we explored the relevance of gene mutations, clinical features, and prognoses to provide evidence for clinical diagnosis and potential treatment strategies.

## Methods

### Patients

Between April 2012 and April 2023, 144 paediatric patients (0–18 years) diagnosed at the Children’s Hospital of Soochow University and who underwent NGS were enrolled. All diagnoses were made according to the MICM criteria. Among them, 97 patients received the Chinese Children’s Leukaemia Group-ALL-2008 (CCLG-ALL-2008) protocol (14) and 47 patients received the Chinese Children’s Cancer Group ALL-2015 (CCCG-ALL-2015) protocol (15). A total of 54 and 90 patients were classified into intermediate-risk (IR) and high-risk (HR) groups, respectively. This study was approved by the Ethics Committee of Children’s Hospital of Soochow University and followed the principles of the Declaration of Helsinki. Written informed consent for participation was obtained from the parents/guardians of all the patients.

### Chemotherapy protocols and risk stratification

The CCLG-ALL-2008 protocol consisted of the following components: Induction therapy: VDLD (vincristine, daunorubicin, L-asparaginase, dexamethasone); Early intensification: CAM ×2 (cyclophosphamide, cytarabine, 6-mercaptopurine); Consolidation: High-dose methotrexate (HD-MTX ×4); Delayed intensification I and II: VDLD plus CAM or D-CAM; Maintenance: 6-mercaptopurine (6-MP) and methotrexate (MTX), with or without additional agents depending on risk group. High-risk patients received intensified treatment modules (HR1–3) adapted from the BFM protocol.

The CCCG-ALL-2015 protocol included: Induction therapy: PVDL (prednisone, vincristine, daunorubicin, L-asparaginase); Early intensification: CAT ×2 (cyclophosphamide, cytarabine, 6-MP); Consolidation: High-dose methotrexate (HD-MTX×4); Continuation (Weeks 16–31): DVDL (dexamethasone, vincristine, daunorubicin, L-asparaginase); Reinduction (Weeks 32–34): High-dose cytarabine (HD-Ara-C); Continuation (Weeks 35–54): 6-MP, MTX, and DVCA (dexamethasone, vincristine, cyclophosphamide, cytarabine); Maintenance: 6-MP and MTX, with or without vincristine and dexamethasone depending on randomised group assignment. High-risk patients under the CCCG-ALL-2015 protocol were eligible for hematopoietic stem cell transplantation.

For T-ALL patients, risk stratification in both protocols was based on a combination of clinical and response-related parameters, including: response to prednisone window therapy, bone marrow morphology on Day 15 (CCLG) or Day 19 (CCCG), and again on Day 33 or 46, initial white blood cell (WBC) count, extramedullary involvement, including central nervous system (CNS) or testicular infiltration, presence of high-risk cytogenetic abnormalities, if available.

### Targeted sequencing and analysis

TRIzo (invitrogen, Cat 15596026) was used to isolate total RNA from bone marrow samples. Ribosomal RNA (rRNA) was depleted, and the stranded RNA-Seq libraries were rapidly constructed from 1 µg of purified total RNA (KAPA RNA HyperPrep Kit with RiboErase (HMR) KIT) according to the manufacturer’s instructions. After fragmentation, cDNA synthesis, adapter ligation, and library amplification, each library was sequenced on an Illumina NovaSeq 6000 platform with a 150-cycle paired-end sequencing. The raw FASTQ data were filtered using the fastp (version 0.23.4) software, which involved the removal of adapter sequences and low-quality sequencing reads. Subsequently, the filtered data were aligned to the reference genome (GRCh37) using STAR (version 2.7.10a) software. The aligned sequences were sorted using the Sambamba (version 0.6.6) software. Duplicate reads in the sorted sequences were removed using the GATK (version 4.0.5.1) software. After filtering, alignment, sorting, and duplicate removal, the resulting BAM files were used for the following analyses: (1) IKZF1 deletions were calculated using rnapeg (version 2.7.7) software; (2) the fusion genes were analysed using Arriba (version 2.4.0), Cicero (version 1.9.6), and Fusioncatcher (version 1.33) software; (3) gene expression levels were quantified using htseq-count (version 2.0.2) software. (4) INDEL analysis: INDELs (insertions and deletions) were detected using the lianti (revision 142) software. The detected mutations were annotated using annovar (updated on 2020-06-08) and snpeff (version 5.1f). All molecular testing was performed on bone marrow samples to ensure detection accuracy, as peripheral blood may contain fewer tumor cells and yield lower variant allele frequency (VAF).

### Statistical analysis

All statistical analyses were performed using SPSS version 25 and R 4.3.2 software. Associations between specific gene mutations and clinical features were analysed using Fisher’s exact test or chi-square test. To account for multiple testing, false discovery rate (FDR) correction was applied. Overall survival (OS) was defined as the time from diagnosis to death. Event-free survival (EFS) was defined as the time from diagnosis (or treatment initiation) to the occurrence of any of the following events: failure to achieve complete remission (refractory disease), relapse, disease progression, or death from any cause. Cumulative incidence of treatment failure (CITF) is defined as the cumulative probability over time of experiencing either relapse or refractory disease, while accounting for death as a competing event. Median follow-up time was calculated using the reverse Kaplan–Meier method, in which censoring and event statuses are reversed. Survival functions were estimated using the Kaplan–Meier method and compared using the log-rank test. Cox regression analysis was used to identify factors associated with OS and EFS. CITF was estimated using a competing risk model and death in complete remission (CR) was treated as a competing event. Gray’s test was used for univariate comparisons between groups. Variables with P < 0.20 in univariate analysis were included in both the Cox proportional hazards model and the multivariable competing risk model. P<0.05 was considered statistically significant.

## Results

### Clinical characteristics of 144 childhood T-ALL

Among the 144 paediatric patients with T-ALL, 116 (80.6%) were male and 28 (19.4%) were female, with a ratio of 4.1:1. The median age at diagnosis was 8.0 years (range 1.2–16.8 years). The median white blood cell (WBC) at diagnosis was 106.33 ×10^9^/L (range 1.05–750.20). The median haemoglobin and platelet counts at diagnosis were 98.50 g/L (range 37.00–159.00) and 63.00×10^9^/L (range 5.00–412.00), respectively. Regarding the immunophenotype, 6 patients had early thymic precursor (ETP) (4.2%), and 138 had non-ETP (95.8%) in our cohort. At the end of follow-up, 64 patients (44.4%) underwent haematopoietic stem cell transplantation (HSCT), while 80 patients (55.6%) received chemotherapy alone. Among the 64 patients who received HSCT, 54 were transplanted in first complete remission (CR1), 9 in second complete remission (CR2) following relapse, and 1 patient underwent transplantation without achieving remission (NR). The baseline patient characteristics are summarised in **Table 1**.

**Table 1:** The clinical characteristics of 144 pediatric T-ALL.

Variables	Total cohort (n=144)
Sex, n (%)	
Male	116 (80.6)
Female	28 (19.4)
Age, n (%)	
≥ 10 years	48 (33.3)
1-9.9 years	96 (66.7)
WBC at diagnosis (×10^9^/L, median, range)	106.33 (1.05-750.20)
Haemoglobin at diagnosis (g/L, median, range)	98.50 (37.00-159.00)
Platelet at diagnosis (×10^9^/L, median, range)	63.00 (5.00-412.00)
Cytogenetic, n (%)	
Normal	74 (51.4)
Hyperdiploid	7 (4.9)
Hypodiploid	4 (2.8)
Pseudodiploid	32 (22.2)
No observation	27 (18.8)
Immunophenotype, n (%)	
ETP	6 (4.2)
Non-ETP	138 (95.8)
Risk stratification, n (%)	
Intermediate risk	54 (37.5)
High risk	90 (62.5)
Fusion gene, n (%)	
SIL::TAL1	34 (23.6)
MLL rearrangement	11 (7.6)
HOX11::rearrangement	11 (7.6)
MTAP-CDKN2B-AS1	11 (7.6)
LOM2::rearrangement	7 (4.9)
NUP214-ABL	5 (3.5)
ETV6 rearrangement	4 (2.8)
HOXA13 rearrangement	4 (2.8)
Negative	76 (52.8)
Treatment, n (%)	
Chemotherapy	80 (55.6)
HSCT	64 (44.4)

WBC, white blood cell; ETP, early thymic precursor. HSCT, hematopoietic stem cell transplantation.

### Gene mutations and signalling pathways in 144 T-ALL cases

**Figure 1:**
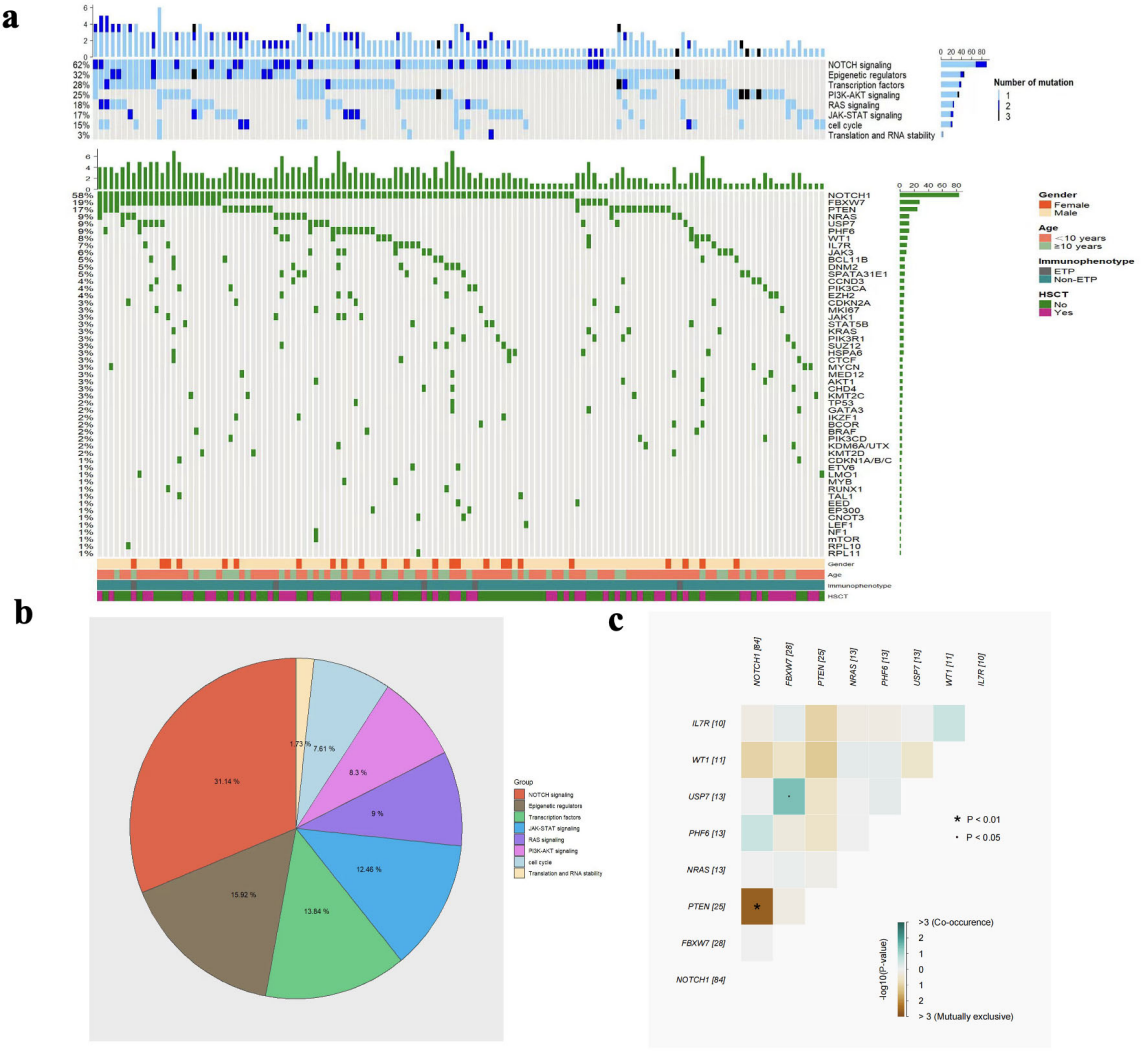
The heatmap of gene mutations and signalling pathways **(a)**, the proportion of each signalling pathway **(b)**, and the correlation analysis of co-occurring gene mutations **(c)**.

The gene mutation data from 144 children with T-ALL who underwent second-generation sequencing were collected. Among them, 142 patients (98.6%) harboured at least one mutation, and 125 patients (86.8%) had two or more mutations. After applying the VAF threshold, we focused our analysis on mutations of potential clinical relevance. NGS results revealed that the top 20 genes were *NOTCH1* (58.3%, 84/144), *FBXW7* (19.4%, 28/144), *PTEN* (17.4%, 25/144), *NRAS* (9%, 13/144), *PHF6* (9%, 13/144), *USP7* (9%, 13/144), *WT1* (6.9%, 10/144), *IL7R* (6.9%, 10/144), *JAK3* (6.3%, 9/144), *EZH2* (4.9%, 7/144), *BCL11B* (4.9%, 7/144), *DNM2* (4.9%, 7/144), *SPATA31E1* (4.9%, 7/144), *CCND3* (4.2%, 6/144), *KRAS* (3.5%, 5/144), *PIK3R1* (3.5%, 5/144), *CDKN2A* (3.5%, 5/144), *STAT5B* (3.5%, 5/144), *SUZ12* (3.5%, 5/144), *MKI67* (3.5%, 5/144) (**Figure 1a**). Next, we divided those gene mutations into eight groups according to the signalling pathways involved in gene mutations and their functions, including: NOTCH (62.5%, 90/144), epigenetic regulators (31.9%, 46/144), transcriptional factors (27.8%, 40/144), PI3K-AKT (25%, 36/144), RAS (18.1%, 26/144), JAK-STAT (16.7%, 24/144), cell cycle regulation (15.3%, 22/144), translation and RNA stability (3.5%, 5/144) pathways (**Figure 1b**). In addition, we analysed the co-occurrence of the mutated genes. As shown in **Figure 1c**, we found pairwise associations between NOTCH1 and PTEN (P<0.01), *FBXW7* and *USP7* (P<0.05).

### The relationship between gene mutations and clinical features

**Figure 2:**
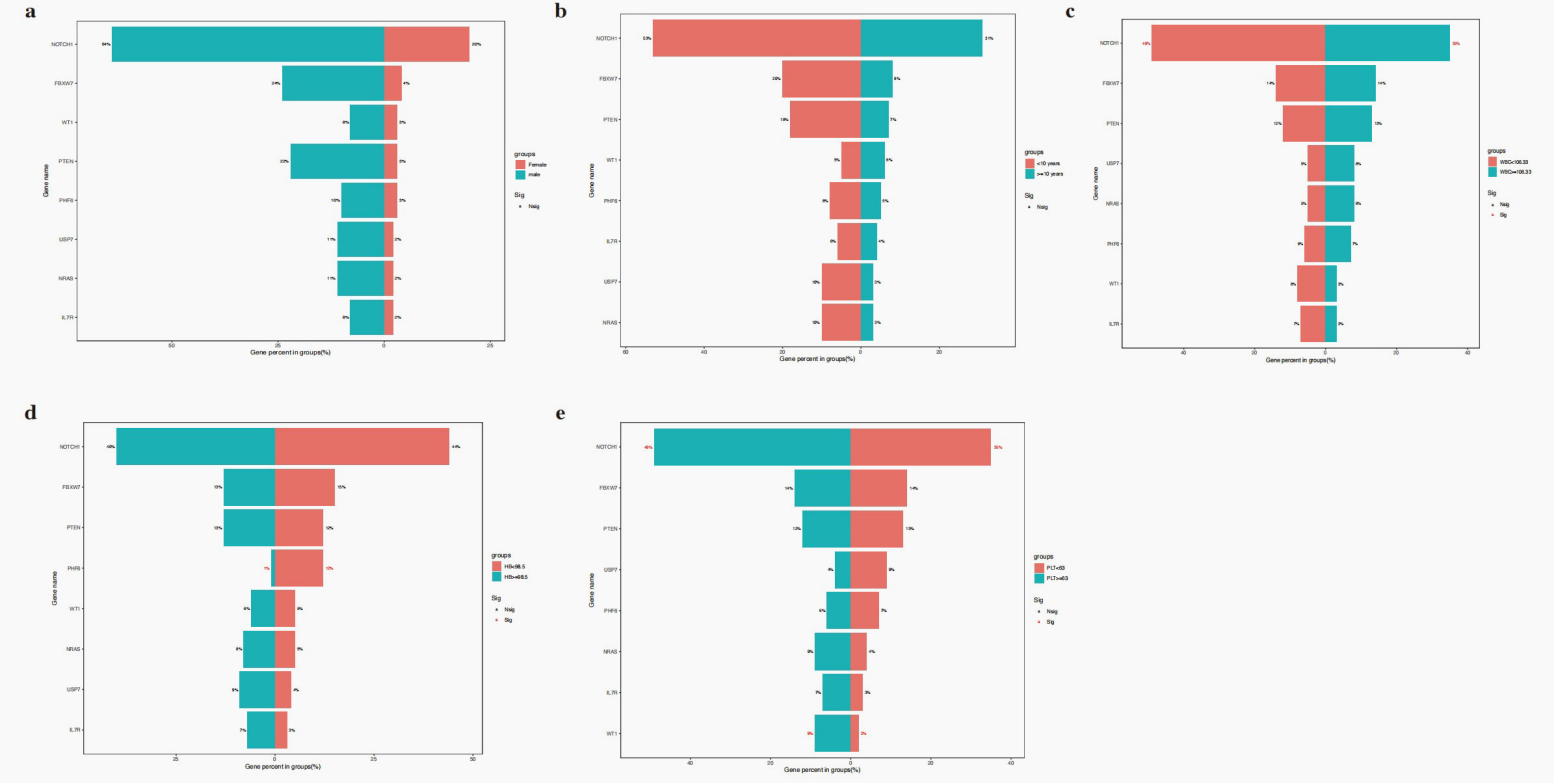
The relationship of gene mutations and sex **(a)**, age **(b)**, WBC **(c)**, haemoglobin **(d)** and platelet **(e)**.

No significant differences in gene mutation were observed between male and female groups (all P>0.050) (**Figure 2a**). No significant differences in gene mutation were observed between 1–9.9 years and ≥10 years groups (all P>0.050) (**Figure 2b**). Moreover, *NOTCH1* mutations were associated with elevated WBC counts (P = 0.018) (**Figure 2c**). Patients with *PHF6* mutations were more likely to develop lower haemoglobin levels at diagnosis (P = 0.001) (**Figure 2d**). Additionally, *NOTCH1* mutations were associated with a higher platelet count at diagnosis (P = 0.030) (**Figure 2e**).

**Figure 3:**
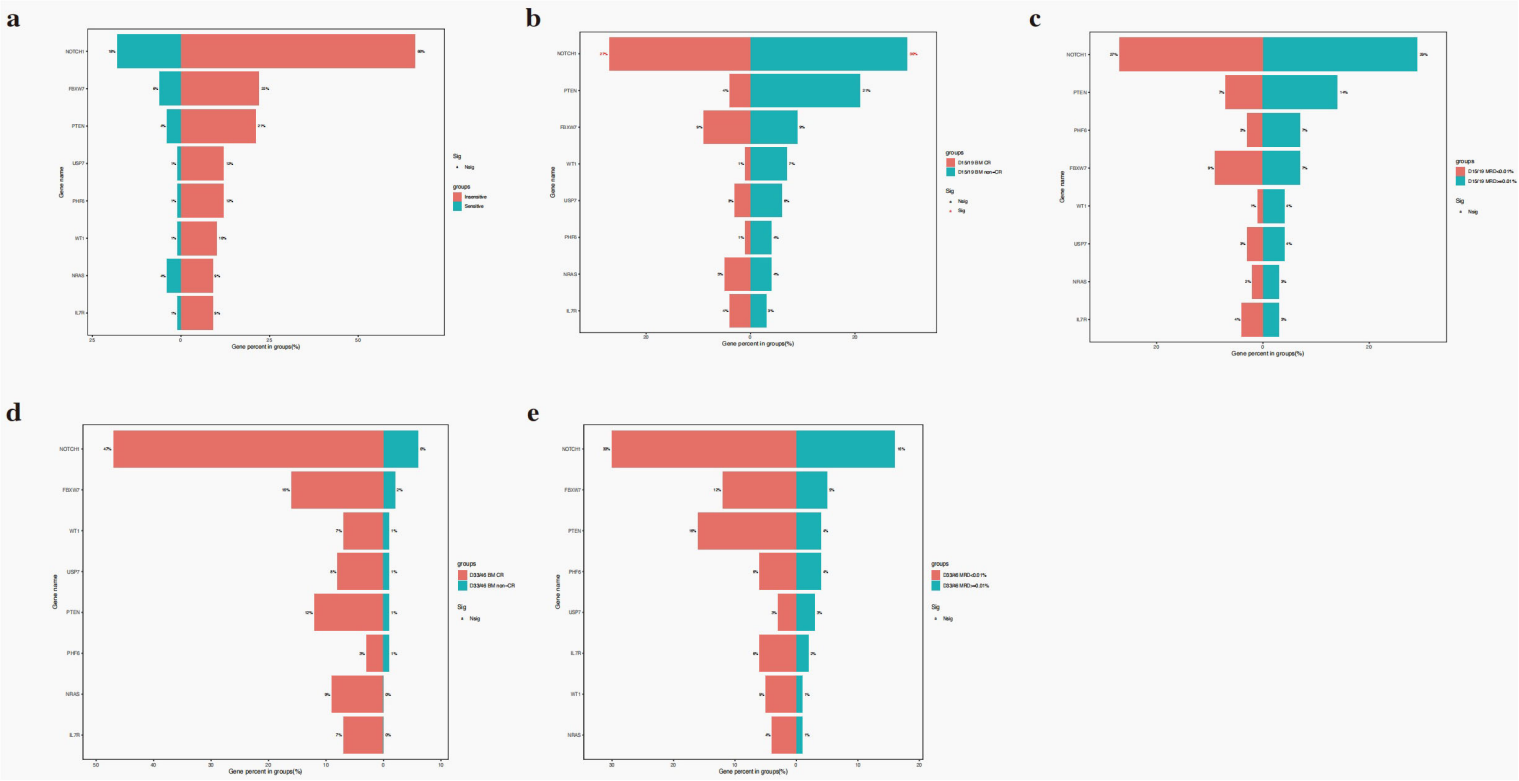
The relationship of gene mutations and steroid response **(a)**, D15/19 BM response **(b)**, D33/46 BM response **(c)**, D15/19 MRD **(d)**, D33/46 MRD **(e)**.

No significant differences in gene mutation were observed between insensitive and sensitive to steroid treatment in this cohort (all P>0.050) (**Figure 3a**). The *NOTCH1* mutation was associated with a better D15/19 BM response with a worse response (P = 0.023, **Figure 3b**). No gene mutations were related to other early treatment outcomes, such as D15/19 minimal residual disease (MRD), D33/46 BM response, and MRD (**Figure 3c–e**).

### The role of gene mutations on the prognosis of paediatric T-ALL

**Figure 4:**
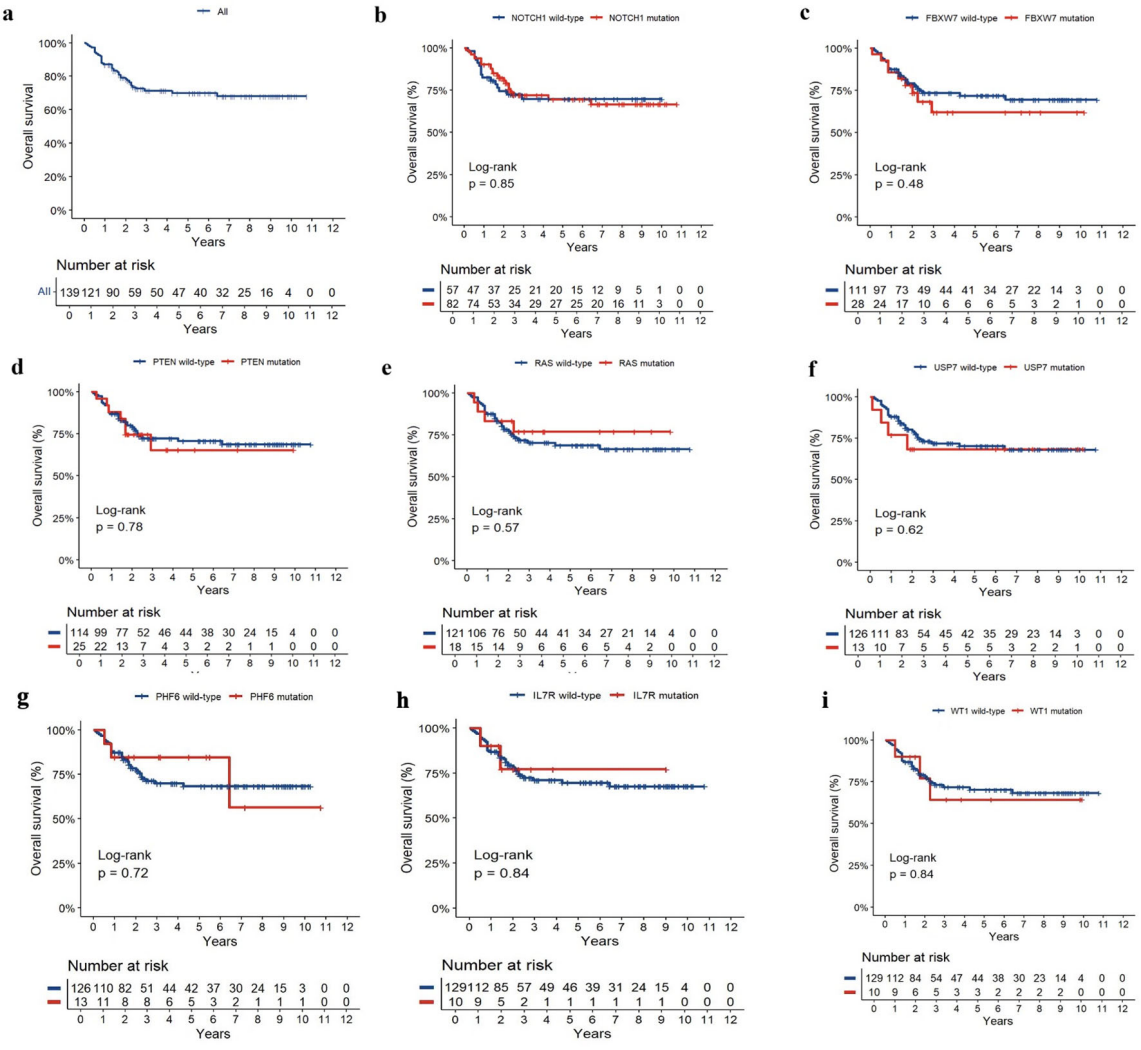
The curves of OS **(a)** and the prognosis of NOTCH1 **(b)**, FBXW7 **(c)**, PTEN **(d)**, RAS (NRAS/KRAS) **(e)**, USP7 **(f)**, PHF6 **(g)**, WT1 **(h)**, and IL7R **(i)** mutation in pediatric T-ALL.

The latest follow-up occurred on 2023 December 31, and the median follow-up was 42 months (range, 1.0–129.0 months). The 5-year OS for 139 paediatric T-ALL was 69.90 ± 4.62% (**Figure 4a**). The ten most common gene mutations were *NOTCH1*, *FBXW7*, *PTEN*, *RAS (NRAS/KRAS)*, *PHF6*, *USP7*, *WT1*, and *IL7R*. We explored their prognostic roles in the 5-year OS of 139 paediatric patients with T-ALL (5 of 144 patients were lost to follow-up). However, no prognostic biomarker was found among the gene mutations that influenced the survival rate of these patients (**Figure 4b–i**).

### The role of *NOTCH1* mutation load in the treatment response, prognosis of childhood T-ALL

**Figure 5:**
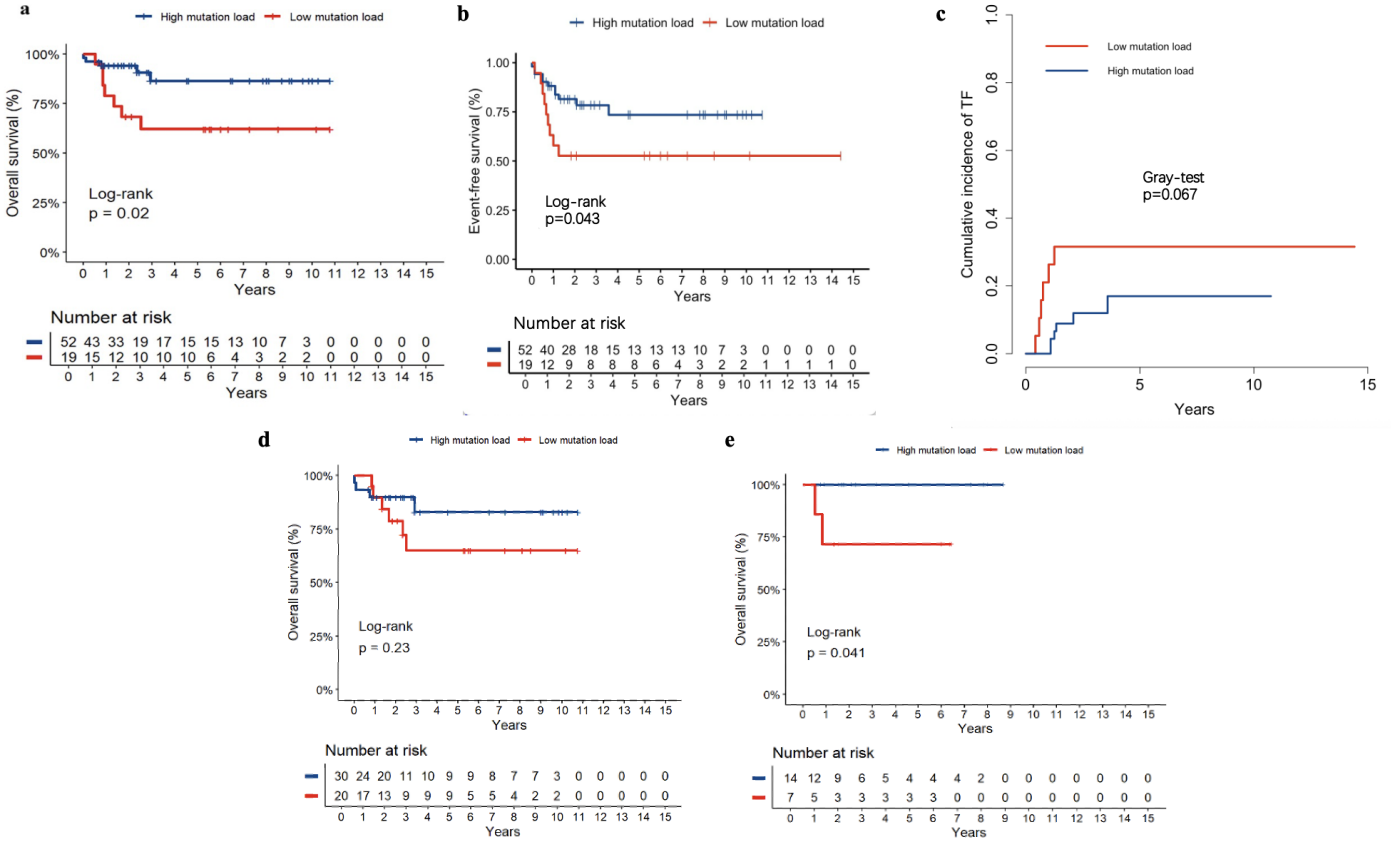
The 5-year OS **(a)**, EFS **(b)** and CITF **(c)** of high and low NOTCH1 mutation load, The role of VAF on the OS of the training set (CCLG-ALL-2008, **(d)** and validation set (CCCG-ALL-2015, **(e)**.

Of 84 patients with *NOTCH1* mutations, 71 had variant allele frequencies (VAF) data. Based on the maximally selected log-rank statistic, a VAF of 12.850 was the cutoff point for differentiating patient prognosis. Patients were categorised into high (52 patients) and low (19 patients) mutation load groups. Regarding clinical characteristics, the high mutation load group had higher bone marrow blasts at diagnosis, and a lower proportion underwent allogeneic HSCT. Other clinical features were not significantly different between the two groups (all P>0.05). The clinical features of the 71 patients are summarised in **Table 2**, and the detailed results with false discovery rate (FDR) correction are provided in **Supplementary Table 1**. Regarding the association between *NOTCH1* mutation load and early treatment response, we found that a high *NOTCH1* mutation load have a lower proportion of MRD ≥1% on D15 (**Table 3**). Additionally, we examined the impact of the *NOTCH1* mutation load on the 5-year OS, EFS and CITF in the cohort of 71 paediatric patients with T-ALL. Our results indicated that patients with a high *NOTCH1* mutation load have higher 5-year OS (**Figure 5a**) and EFS (**Figure 5b**) than those with a low *NOTCH1* mutation load (P = 0.02 and 0.043, respectively). No statistically significant difference was observed for CITF between the two groups (**Figure 5c**), although a trend toward lower incidence was noted in the high mutation load group (Gray’s test, P = 0.067). **Table 4** presents the univariate and multivariate analyses of the factors affecting the 5-year OS, EFS, and CITF. In the univariate analyses, we found that platelet count less than and equal to 50×10^9^ was a risk factor for 5-year OS, BM not CR after induction was a risk factor for 5-year EFS, while a high *NOTCH1* mutation load was a protective factor for 5-year OS and EFS. In the multivariate analyses, a high *NOTCH1* mutation load was an independent protective factor for the 5-year OS (P = 0.042) and 5-year EFS (P = 0.0104). However, high VAF was not significantly associated with CITF in our univariate and multivariate analysis. We further validated the role of VAF in *NOTCH1* mutations on paediatric T-ALL OS. The training set consisted of 50 patients treated with the CCLG-ALL-2008 protocol, and 21 patients who received the CCCG-ALL-2015 protocol were used for the validation set. A VAF of 0.1594 was identified as the cutoff point for differentiating prognosis among patients treated with CCLG-ALL-2008 protocol (83.0 ± 8.39 vs 65.0 ± 11.82, P = 0.23, **Figure 5c**). When this cutoff was applied to patients who received CCCG-ALL-2015, we still found superior survival in the high mutation load group (100% vs 71.4 ± 17.1%, P = 0.041, **Figure 5d**).

**Table 2:** The clinical characteristics of 52 high mutation load and 19 low mutation load patients.

Variables	High mutation load(n=52)	Low mutation load(n=19)	P value
Age at diagnosis, median (range), years			0.274
≥10	18	4	
<10	34	15	
Sex			0.079
Male	25	19	
Female	14	3	
WBC at diagnosis, median (range), ×10^9^/L	100.54 (1.05-693.82)	136.69 (25.68-539.76)	0.199
Haemoglobin at diagnosis, median (range), g/L	95.00 (37.00-159.00)	101.00 (46.00-155.00)	0.631
PLT at diagnosis, median (range), ×10^9^/L	76.50 (8.00-412.00)	51.00 (15.00-196.00)	0.455
BM blasts (%)	89.00 (62.00-98.00)	84.00 (75.00-97.00)	0.026*
Immunophenotype			1.000
ETP	2	1	
Non-ETP	49	18	
CNSL			1.000
Yes	1	0	
No	50	19	
SIL::TAL1			1.000
Yes	12	5	
No	39	14	
Risk stratification			0.761
Intermediate risk	24	8	
High risk	28	11	
HSCT			0.024*
Yes	15	11	
No	37	8	

WBC, white blood cell; PLT, platelet; BM, bone marrow; ETP, early thymic precursor; CNSL, central nervous system leukaemia; HSCT, hematopoietic stem cell transplantation.Bold values and * indicate statistical significance (p < 0.05).

**Table 3:** The relationship between NOTCH1 mutation load and early treatment response.

	High mutation load (n=52)	Low mutation load (n=19)	P value
Steroid response (n=65)			0.667
Good	12	3	
Poor	35	15	
D15/19 BM (n=65)			0.519
CR	26	8	
PR	10	5	
NR	10	6	
D33/46 BM (n=62)			1.000
CR	38	15	
PR	5	2	
NR	2	0	
D15/19 MRD (n=42)			0.004*
<0.01%	23	1	
≥0.01 to <1%	3	2	
≥1%	7	6	
D33/46 MRD (n=40)			0.095
<0.01%	22	5	
≥0.01 to <1%	4	4	
≥1%	5	0	

BM, bone marrow; CR, complete remission; PR, partial remission; NR, no remission; MRD, Measurable residual disease.Bold values and * indicate statistical significance (p < 0.05).

**Table 4:** The results of univariable and multivariable cox regression model for 5-years OS and EFS, competing risk regression model for 5-years CIR in childhood T-ALL.

Factor	5-year OS				5-year EFS				5-year CITF			
	Univariable analysis		Multivariable analysis		Univariable analysis		Multivariable analysis		Univariable analysis		Multivariable analysis	
	HR (95% CI)	P value	HR (95% CI)	P value	HR (95% CI)	P value	HR (95% CI)	P value	HR (95% CI)	P value	HR (95% CI)	P value
Male vs female	0.811 (0. 411–1.600)	0.545			1.559 (0.513–4.737)	0.434			1.118 (0. 355–3.525)	0.850		
Age at diagnosis (≥10 years vs<10 year)	0.723 (0.366–1.427)	0.349			0.410 (0.119–1.419)	0.159	0.564 (0.158-2.015)	0.378	0.319 (0.072–1.409)	0.130	0.314 (0.073–1.350)	0.120
WBC≥100×10^9^/L	2.740 (0.740–10.139)	0.131	0.663 (0.116–3.781)	0.644	1.446 (0.560–3.739)	0.446			1.290 (0.476–3.493)	0.620		
Haemoglobin ≤ 100g/L	0.738 (0.238–2.293)	0.599			1.304 (0.505–3.886)	0.573			1.185 (0.427–3.292)	0.740		
Platelet ≤ 50×10^9^	3.419 (1.029–11.362)	0.045*	2.265 (0.540–9.504)	0.264	1.328 (0.524–3.366)	0.550			1.946 (0.712–5.322)	0.190	2.068 (0.768–5.568)	0.150
BM blasts≥90%	1.176 (0.313–4.427)	0.810			0.748 (0.266–2.097)	0.580			0.453 (0.137–1.503)	0.210		
ETP vs non-ETP	1.764 (0.227–13.690)	0.587			0.974 (0.130–7.332)	0.978			1.360 (0.153–12.083)	0.780		
SIL-TAL1 yes/no	1.234 (0.333–4.568)	0.753			0.726 (0.210–2.509)	0.613			0.568 (0.123–2.636)	0.470		
HOX11L2 yes/no	0.600 (0.145–2.479)	0.480			1.327 (0.305–5.778)	0.706			0.716 (0.115–4.482)	0.720		
MLL-r yes/no	1.209 (0.293–4.997)	0.793			1.144 (0.152–8.600)	0.896			1.411 (0.214–9.302)	0.720		
CNSL yes/no	2.125 (0.656–6.888)	0.209			1.270 (0.415–3.886)	0.675			1.220 (0.153–9.735)	0.850		
Steroid response poor vs good	4.002 (0.507–31.580)	0.188	3.195 (0.345–29.611)	0.307	2.539 (0.583–11.061)	0.215			1.216 (0.346–4.270)	0.760		
BM not CR after induction	1.321 (0.153–11.399)	0.800			4.915 (1.410–17.132)	0.012	6.040 (1.570–23.23)	0.0089*	2.074 (0.508–8.470)	0.310		
MRD after induction (positive vs negative)	7.150 (0.784–65.170)	0.081	NA^§^	NA	3.241 (0.909–11.560)	0.07	NA	NA	1.642 (0.362–7.437)	0.520		
High mutation load	0.278 (0.088–0.878)	0.029*	0.246 (0.064–0.951)	0.043*	0.324 (0.128–0.817)	0.017*	0.281 (0.106-0.742)	0.0104*	0.395 (0.141–1.107)	0.067	0.460 (0.164–1.287)	0.140

HR, hazard ratio; WBC, white blood cell; BM, bone marrow; ETP, early thymic precursor; MLL-r, MLL rearrangement; CNSL, central nervous system leukaemia; CR, complete remission; HSCT, hematopoietic stem cell transplantation.Bold values and * indicate statistical significance (p < 0.05).

## Discussion

In this study, we report a spectrum of gene mutations and their clinical features and prognostic roles in paediatric patients with T-ALL. *NOTCH1* mutation (58.3%) and *NOTCH1* signalling pathway (62.5%) were the most common gene mutations and pathways in this study, respectively. Additionally, we found that certain gene mutations were associated with different clinical features in children with T-ALL. However, we did not identify any genetic mutations that could influence the prognosis of paediatric patients with T-ALL.

Several studies have investigated the clinical characteristics of various genetic mutations. In adult patients with T-ALL, Asnafi et al. found that the *NOTCH1* mutation group had lower WBC counts than the wild-type group (16). Our results are consistent with these findings. We observed that NOTCH1 mutations were associated with higher platelet counts. Steroid response is recognised as a strong predictor of treatment response and outcomes in children with ALL (17, 18). The most commonly reported genetic mechanism associated with the steroid response in paediatric T-ALL is the activation of IL-7R signalling. A study published in *Leukemia* found that IL-7R mutations contribute to steroid resistance in children with T-ALL (19). However, our study did not observe such an association between IL-7R mutation and steroid response, and this discrepancy warrants further investigation.

Mutations in *NOTCH1* and *FBXW7* result in the abnormal activation of the *NOTCH1* signalling pathway, which is crucial in the pathogenesis of T-ALL (20). Previously, the reported incidence rate of *NOTCH1* mutation in pediatric T-ALL was more than 50% (21, 22). In this cohort, 58.3% of paediatric patients with T-ALL had *NOTCH1* mutations, and 19.4% had *FBXW7* mutations. Several studies suggested that *NOTCH1* or *FBXW7* mutations may predict a better outcome in pediatric T-ALL (8, 9, 23). Recently, a large cohort of 1300 childhood T-ALL cases indicated that most *NOTCH1* variants, except intronic single nucleotide variant and intragenic losses, were associated with superior OS and EFS (24). While *NOTCH1* or *FBXW7* mutations were related to superior survival in most studies, they were not correlated with survival in this study. We speculate that this difference may be attributable to the diversity of treatment strategies across different centres. Most patients with *NOTCH1* mutations at our centre underwent HSCT (37 of 84, 44%), which may account for the favourable role of this mutation. In another centre with the same treatment protocol, the survival rate of patients with *NOTCH1* mutation did not significantly increase (25). Therefore, the diversity of protocols from different regions in paediatric T-ALL may have contributed to this difference. More high-quality studies are warranted to investigate the prognostic role of *NOTCH1* or *FBXW7* mutations in paediatric patients with T-ALL, particularly in China.

PTEN is one of the most frequently expressed oncosuppressors and is commonly inactivated in human cancers, including T-ALL (26). It is a negative regulator of the phosphatidylinositol-3 kinase (PI3K)/Akt or mechanistic target of rapamycin (mTOR) signalling pathway (27). In previous studies, *PTEN* mutations were detected in 11–27% of pediatric T-ALL cases (28). However, the prognostic value of *PTEN* in paediatric T-ALL remains unclear. In a cohort from the MRC UKALL2003 trial, the prognoses of patients with PTEN abnormalities and the wild-type group were not significantly different (29). Our results align with these findings. However, among children with T-ALL treated with ALL IC-BFM protocols, *PTEN* mutation was associated with poor treatment response and unfavourable clinical outcomes (30). Pölönen et al. reported that deletions or losses were associated with worse outcomes (24). The role of *PTEN* in T-ALL requires further investigation and subdivision.

RAS genes are members of the small GTPase family and include three separate genes: *NRAS*, *KRAS*, and *HRAS* (31). The reported incidence rates of *NRAS* and *KRAS* were 9.38% and 3.13% in a cohort of 64 Korean children with T-ALL (6). Similar results were observed in our 144 paediatric T-ALL cohort, with *NRAS* and *KRAS* present at 9.0% and 3.5%, respectively. Perentesis et al. indicated that *RAS* mutations were present in approximately 15% of paediatric cases and were not associated with inferior outcomes in 870 children with ALL (32). In this study, the survival rates of patients with *RAS* mutations were consistent with their results. However, the prognostic role of *RAS* mutations in paediatric T-ALL has rarely been investigated, is inconsistent, and needs further exploration. Van Vlierberghe et al. indicated that paediatric and adult patients with T-ALL, with or without *PHF6* mutations, did not influence the overall outcome (33), which is consistent with our results.

Changes in VAF resulting from different gene mutations play an important role in predicting disease status and assessing prognosis in adult myelodysplastic syndrome and acute myeloid leukaemia (34). Zhang et al. reported that a high burden of *IKZF1* mutations (VAF>0.20) is associated with inferior OS in AML (35). Another study indicated that *TP53* VAF>40% is an independent risk factor for lower OS in patients with myelodysplastic syndromes (MDS) (36). These studies emphasised the role of VAF in the prognosis of haematologic malignancies, whereas few studies have explored its significance in ALL, especially T-ALL. Our results indicated that a higher *NOTCH1* mutation load was associated with better D15 response and superior survival compared to a lower mutation load, emphasising the role of VAF in patients with *NOTCH1* mutation in T-ALL. However, the limited number of patients necessitates large-scale, high-quality prospective studies to further explore the role of VAF in paediatric T-ALL.

This study has limitations. First, the long accrual period and use of different treatment protocols may have introduced heterogeneity that affects the precision of our estimates. Second, as a retrospective analysis with a relatively small sample size, it is susceptible to selection and information bias. Third, MRD status was unavailable for many patients, precluding robust evaluation of its association with prognosis. Fourth, VAF of this study was derived from RNA sequencing data, which can be influenced by both the proportion of mutant transcripts and gene expression levels, making it challenging to disentangle these effects. Fifth, as only RNA NGS was performed, some diagnostic mutations may have been missed. Finally, the assessed links between gene mutations and clinical features were exploratory and underpowered; thus, they should be regarded as hypothesis-generating rather than definitive. Confirmation in larger, independent cohorts is required, and well-designed, multicentre prospective studies are warranted to further clarify how clinical features and diverse genetic alterations relate to outcomes.

In conclusion, several correlations were found between clinical features and gene mutations. Higher VAF in *NOTCH1* mutations with superior survival rates were detected in our study. Using NGS technology analysis can improve the accurate prediction of risk stratification and prognosis in paediatric T-ALL, helping to identify potential targets for immunotherapy and guiding treatment to improve the long-term outcomes of children with T-ALL.

## Data Availability

The raw data supporting the conclusions of this article will be made available by the authors, without undue reservation.
