# Supplementary figures and images for: Next-generation sequencing reveals clinical features and prognosis of gene mutations in Chinese children with T-cell acute lymphoblastic leukaemia
Source: Front Oncol. 2025 Sep 26;15:1666527. doi: 10.3389/fonc.2025.1666527 (PMC12510939; doi:10.3389/fonc.2025.1666527)

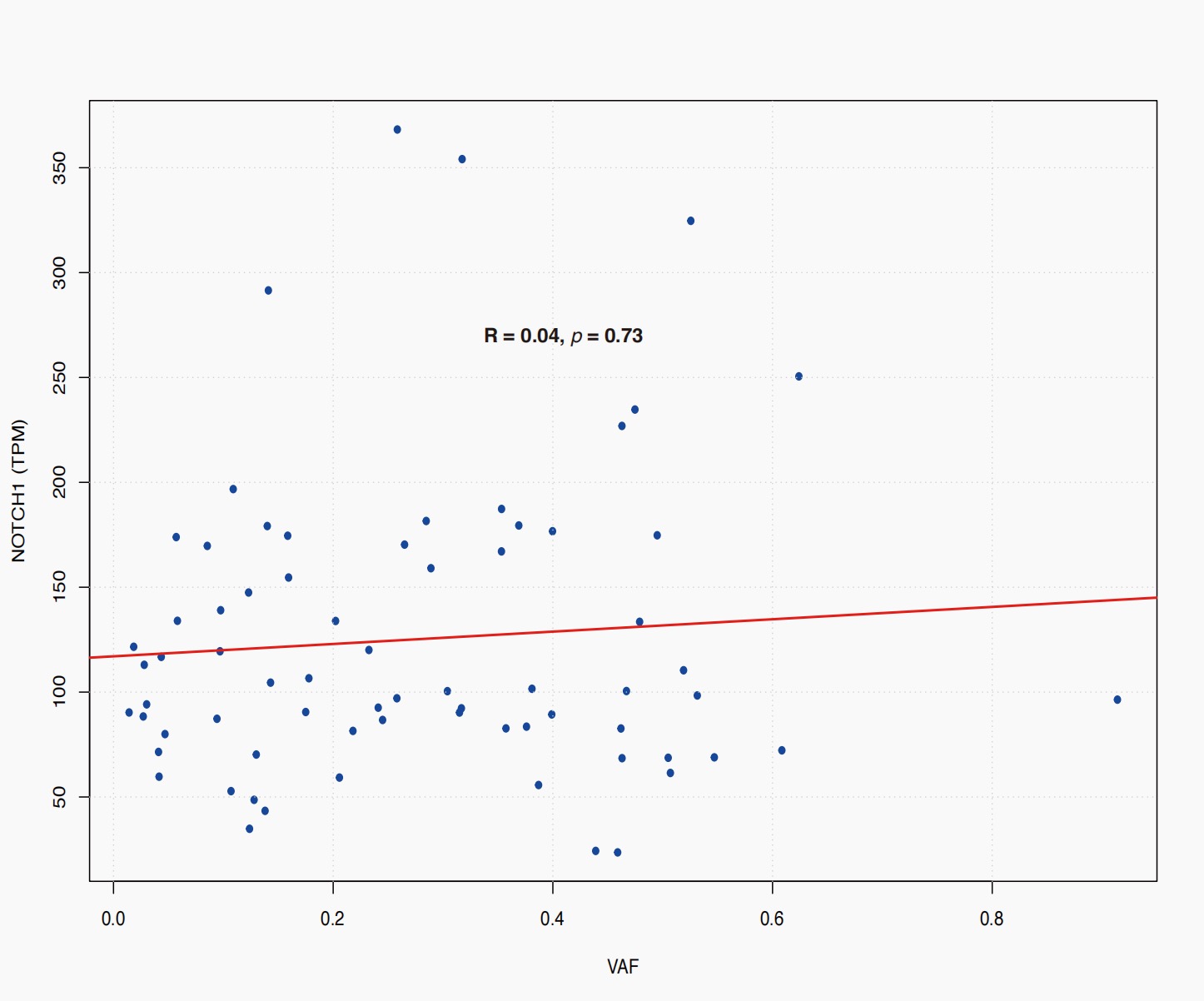

Supplement: Supplementary file 1 [file Image1.jpeg]
