# Supplementary material for: Next-generation sequencing reveals clinical features and prognosis of gene mutations in Chinese children with T-cell acute lymphoblastic leukaemia
Source: Front Oncol. 2025 Sep 26;15:1666527. doi: 10.3389/fonc.2025.1666527 (PMC12510939; doi:10.3389/fonc.2025.1666527)
Supplement: Supplementary file 2 [file Table1.docx]

Supplemental Table 1: The clinical characteristics of 52 high mutation load and 19 low mutation load patients (include FDR-adjusted P values).

| **Variables** | **High mutation load(n=52)** | **Low mutation load(n=19)** | **P value** | **Adjusted P value** |
| --- | --- | --- | --- | --- |
| Age at diagnosis, median (range), years |  |  | 0.274 | 0.603 |
| ≥10 | 18 | 4 |  |  |
| ＜10 | 34 | 15 |  |  |
| Sex |  |  | 0.079 | 0.290 |
| Male | 25 | 19 |  |  |
| Female | 14 | 3 |  |  |
| WBC at diagnosis, median (range), ×10^9^/L | 100.54 (1.05-693.82) | 136.69 (25.68-539.76) | 0.199 | 0.547 |
| Hemoglobin at diagnosis, median (range), g/L | 95.00 (37.00-159.00) | 101.00 (46.00-155.00) | 0.631 | 0.992 |
| PLT at diagnosis, median (range), ×10^9^/L | 76.50 (8.00-412.00) | 51.00 (15.00-196.00) | 0.455 | 0.834 |
| BM blasts (%) | 89.00 (62.00-98.00) | 84.00 (75.00-97.00) | **0.026*** | **0.143** |
| Immunophenotype |  |  | 1.000 | 1.000 |
| ETP | 2 | 1 |  |  |
| Non-ETP | 49 | 18 |  |  |
| CNSL |  |  | 1.000 | 1.000 |
| Yes | 1 | 0 |  |  |
| No | 50 | 19 |  |  |
| SIL-TAL1 |  |  | 1.000 | 1.000 |
| Yes | 12 | 5 |  |  |
| No | 39 | 14 |  |  |
| Risk stratification |  |  | 0.761 | 1.000 |
| Intermediate risk | 24 | 8 |  |  |
| High risk | 28 | 11 |  |  |
| HSCT |  |  | **0.024*** | **0.143** |
| Yes | 15 | 11 |  |  |
| No | 37 | 8 |  |  |

Abbreviations: *WBC:* white blood cell; *PLT:* platelet; *BM:* bone marrow; *ETP:* early thymic precursor; *CNSL:* central nervous system leukemia; *HSCT:* hematopoietic stem cell transplantation
